# Epistatic interactions between mutations of TACI (*TNFRSF13B*) and *TCF3* result in a severe primary immunodeficiency disorder and systemic lupus erythematosus

**DOI:** 10.1038/cti.2017.41

**Published:** 2017-10-20

**Authors:** Rohan Ameratunga, Wikke Koopmans, See-Tarn Woon, Euphemia Leung, Klaus Lehnert, Charlotte A Slade, Jessica C Tempany, Anselm Enders, Richard Steele, Peter Browett, Philip D Hodgkin, Vanessa L Bryant

**Affiliations:** 1 Department of Virology and Immunology, Auckland City Hospital, Auckland, New Zealand; 2 Department of Clinical Immunology, Auckland City Hospital, Auckland, New Zealand; 3 Cancer Society Research Centre, University of Auckland, Auckland, New Zealand; 4 School of Biological Sciences, University of Auckland, Auckland, New Zealand; 5 Department of Immunology, Walter and Eliza Hall Institute of Medical Research, Parkville, VIC, Australia; 6 Department of Medical Biology, University of Melbourne, Parkville, VIC, Australia; 7 Department of Allergy and Clinical Immunology, Royal Melbourne Hospital, Parkville, VIC, Australia; 8 Department of Immunology and Infectious Disease, John Curtin School of Medical Research and Centre for Personalised Immunology, Australian National University, Canberra, ACT, Australia; 9 Department of Hematology, LabPlus, Auckland City Hospital, Auckland, New Zealand; 10 Department of Molecular Medicine, and Pathology University of Auckland, Auckland, New Zealand

## Abstract

Common variable immunodeficiency disorders (CVID) are a group of primary immunodeficiencies where monogenetic causes account for only a fraction of cases. On this evidence, CVID is potentially polygenic and epistatic although there are, as yet, no examples to support this hypothesis. We have identified a non-consanguineous family, who carry the C104R (c.310T>C) mutation of the Transmembrane Activator Calcium-modulator and cyclophilin ligand Interactor (TACI, *TNFRSF13B*) gene. Variants in *TNFRSF13B*/TACI are identified in up to 10% of CVID patients, and are associated with, but not solely causative of CVID. The proband is heterozygous for the *TNFRSF13B*/TACI C104R mutation and meets the Ameratunga *et al.* diagnostic criteria for CVID and the American College of Rheumatology criteria for systemic lupus erythematosus (SLE). Her son has type 1 diabetes, arthritis, reduced IgG levels and IgA deficiency, but has not inherited the *TNFRSF13B*/TACI mutation. Her brother, homozygous for the *TNFRSF13B*/TACI mutation, is in good health despite profound hypogammaglobulinemia and mild cytopenias. We hypothesised that a second unidentified mutation contributed to the symptomatic phenotype of the proband and her son. Whole-exome sequencing of the family revealed a *de novo* nonsense mutation (T168fsX191) in the Transcription Factor 3 (*TCF3*) gene encoding the E2A transcription factors, present only in the proband and her son. We demonstrate mutations of *TNFRSF13B*/TACI impair immunoglobulin isotype switching and antibody production predominantly via T-cell-independent signalling, while mutations of *TCF3* impair both T-cell-dependent and -independent pathways of B-cell activation and differentiation. We conclude that epistatic interactions between mutations of the *TNFRSF13B*/TACI and *TCF3* signalling networks lead to the severe CVID-like disorder and SLE in the proband.

Common variable immunodeficiency disorders (CVID) are a heterogeneous group of conditions characterised by defective antibody production associated with frequent infections, autoimmunity, chronic inflammation and malignancy.^1^ The dominant feature is late onset antibody failure resulting in immune system failure.^2^ The genetic basis of CVID is currently being studied and is proving complex. Over 12 monogenic defects causing CVID-like disorders have been identified,^3, 4, 5, 6, 7^ most which appear to directly impair B-cell function. Currently if a single causative mutation is identified, by definition such patients are reclassified with a specific molecular diagnosis, for example, NFκB1-deficiency (OMIM CVID12)^6, 7^ and are deemed to have CVID-like disorders. Identification of the genetic basis of primary immunodeficiency disorders has many clinical advantages^9, 10^ including accurate, early diagnosis of mildly symptomatic individuals or those with atypical presentations. This may prompt timely interventions including immunoglobulin (Ig) replacement, to reduce disabling sequelae. If the causative gene defect has been identified in a family, it will allow genetic counselling as well as preimplantation genetic diagnosis, prenatal diagnosis using chorionic villus sampling or amniocentesis.^8, 9^

In contrast, mutations in other genes such as *TNFRSF13B*, which encodes Transmembrane Activator Calcium Modulator and Cyclophilin Ligand Interactor (TACI), MutS homolog 5 (*MSH5*) and *TNFRSF13C*, which encodes B-cell activating factor receptor (BAFFR), predispose to, but do not solely underlie CVID. Mutations of *TNFRSF13B/*TACI are also found in healthy individuals, albeit at lower frequency than in CVID cohorts: the frequency of normal individuals carrying the C104R variant (0.8%) is higher than the prevalence of disease (0.002%).^10^ This suggests either these mutations of TACI are clinically inconsequential in many cases, or there are other additional unknown genetic defects in symptomatic patients that contribute to the disease phenotype.^11, 12^

Here, we report the first example of digenic inheritance leading to a severe CVID-like disorder and autoimmunity, as a result of epistasis. Epistasis, where two or more genetic loci interact to produce novel phenotypes was first predicted over one hundred years ago. However, its existence in humans has been highly controversial because of the scarcity of well-characterised examples.^13, 14, 15^ In this report, superimposition of a *de novo* Transcription Factor 3 (*TCF3*) mutation in a family already carrying a C104R (c.310T>C) mutation of the TACI gene causes a severe CVID-like disorder and systemic lupus erythematosus (SLE) in the proband. Her symptomatic son, who has inherited only the *TCF3* mutation, but not the TACI gene mutation, has type 1 diabetes (T1D), synovitis, reduced IgG levels and IgA deficiency. Other family members, carrying only the TACI mutation, in heterozygous or homozygous form, are either in good health or only present with mild clinical symptoms. Our studies indicate the *TCF3* mutation has a much greater clinical impact than the *TNFRSF13B*/TACI mutation on disease severity and expression of both mutations in the proband results in a severe disorder. The phenotypic pattern of the immunodeficiency and autoimmune disease in this family exemplifies how digenic inheritance can lead to clinical and genetic epistasis in humans.^16^

## Results

### Clinical presentation of index patient

**Figure 1:**
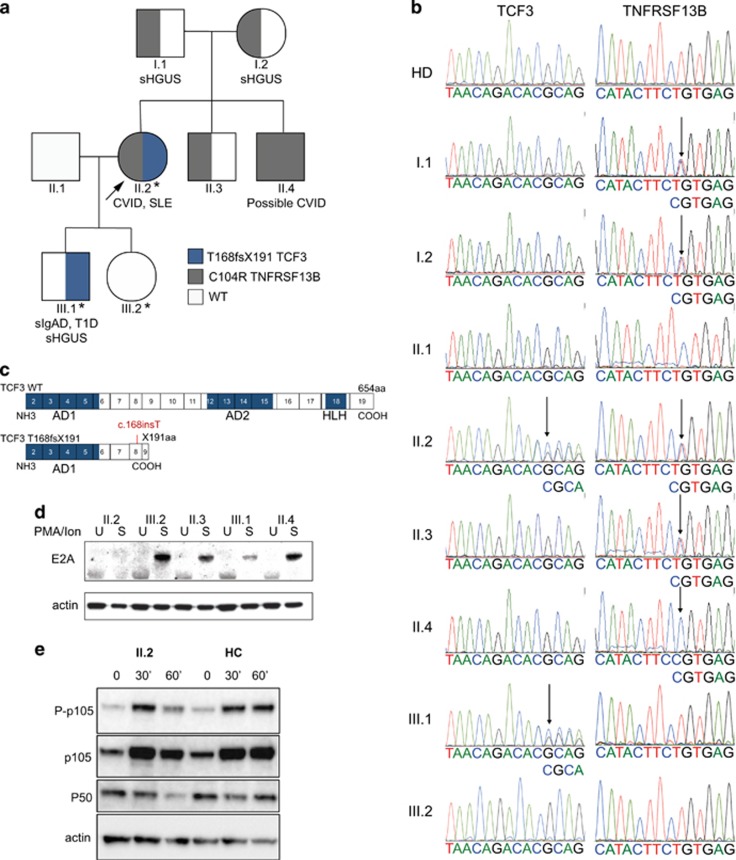
Novel *de novo TCF3* mutation discovered in a CVID family carrying C104R TACI variant. (**a**) Digenic inheritance of *TNFRSF13B* (c.310T>C, C104R TACI) and *TCF3* (T168fx191) mutations in a three-generation New Zealand family. Whole-exome sequencing was performed on II.2, III.1 and III.2 (indicated by *). The proband (II.2) is indicated by an arrow. Circles, female; squares, male; gray, *TNFRSF13B*/TACI C104R mutation; blue *TCF3* T168fsX191 mutation (as indicated). The proband (arrow, II.2) is heterozygous for both the *TCF3* T168fsX191 and *TNFRSF13B*/TACI C104R mutations. Other family members who have inherited *TCF3* T168fsX191 and *TNFRSF13B*/TACI C104R mutations are shown. CVID, common variable immunodeficiency disorder; SLE, systemic lupus erythematosus; sIgAD, selective IgA deficiency; T1D, Type 1 Diabetes, sHGUS, symptomatic hypogammglobulinaemia of uncertain significance; WT, wild-type. (**b**) Electropherograms showing the T168fsX191 mutation of *TCF3* and C104R (c.310T>C) mutation of TACI gene in the proband II.2. The proband’s son (III.1) has inherited the *TCF3* T168fsX191 mutation, but not the *TNFRSF13B*/TACI C104R mutation. The proband’s clinically unaffected daughter (III.2) has not inherited either mutation. The TCF3 T168fsX191 mutation was absent in the proband’s parents, indicating a *de novo* origin. (**c**) Schema of wild-type and truncated mutant *TCF3* T168fsX191 gene. Exons coding E2A functional domains, activation domain 1 and 2 (AD1, AD2) and helix-loop-helix (HLH) domains are shown. (**d**) E2A (E47) protein expression was assessed by western blotting of lysates following 30 min PMA/ionomycin stimulation of PBMCS in the kindred, as indicated (U, unstimulated; S, stimulated). (**e**) PBMCs from the proband (II.2) and healthy control (HC) individuals (*n*=2) were unstimulated, or stimulated with PMA+ionomycin for 30 or 60 min as indicated, and cell lysates were analysed for p105 phosphorylation (P-p105, Ser933), expression of p105 and p50 by western blotting. Beta-actin was used as a protein loading control. Results are representative of two independent experiments.

The proband (II.2), aged 61 years presented with symptomatic hypogammaglobulinemia in her teenage years and was diagnosed with CVID at age 33 (Table 1, Figures 1a and b). She was initially treated with intravenous immunoglobulin, but later changed to subcutaneous immunoglobulin treatment. She has had two episodes of meningitis while receiving immunoglobulin and has chronic diarrhoea. Despite several functional endoscopic sinus surgical procedures, she continues to suffer recurrent upper respiratory tract infections. She is on thyroxine replacement for Hashimoto’s thyroiditis and also meets the American College of Rheumatology (ACR) criteria for SLE. She has cytopenias, antinuclear antibodies, rashes, oral ulcers, nasal ulcers and arthritis.

### Clinical features and segregation of the *TNFRSF13B*/TACI C104R mutation in the kindred

The proband was shown to be heterozygous for the C104R (c.310T>C) mutation of the *TNFRSF13B*/TACI gene in a previous study.^12^ Her non-consanguineous parents (I.1 and I.2), in their ninth and tenth decades, are both heterozygous for the C104R mutation (Figure 1a). They have mild symptomatic hypogammaglobulinemia and thrombocytopenia, but are in otherwise reasonable physical health.^12^ Both the proband’s male siblings carry the C104R mutation and are well. Both have mild cytopenias (Table 1). One brother is heterozygous (II.3) and the other (II.4) is homozygous for the *TNFRSF13B*/TACI C104R mutation.^12^ Given his asymptomatic status, the proband’s brother with the TACI C104R homozygous mutation (II.4) does not meet the Ameratunga *et al.*^17^ criteria for probable CVID; he has normal, albeit transient vaccine challenge responses despite being profoundly hypogammaglobulinemic (IgG 1.6 g l^−1^, NR 7–14; Table 1).^12, 18^ He has declined immunoglobulin replacement and remains in excellent health.

Neither of the proband’s children carry the TACI C104R mutation (Figure 1b, Table 1). The proband’s daughter (III.2) is in good health. The proband’s son (III.1) has CVID-related phenotypes including symptomatic IgG deficiency, IgA deficiency, type 1 diabetes and has recently developed seronegative arthritis. He has high titres of anti-parietal cell antibodies. His disorder may be in evolution as his IgG has decreased to 5.5 g l^−1^ (NR 7–14) from 6.5 g l^−1^ over the last year. He suffers from recurrent infections and has impaired antigen responses to protein and carbohydrate vaccines (Table 1). He is classified as having symptomatic hypogammaglobulinemia of uncertain significance and IgA deficiency.^17, 19^ Both the proband and her son have reduced switched memory B cells and the proband is lymphopaenic (Table 1). Since symptomatic disease is a prerequisite for probable CVID, application of our CVID diagnostic criteria^17, 19^ concluded that only the proband (II.2) had CVID, while her son (III.1) had reduced IgG levels (symptomatic hypogammaglobulinemia of uncertain significance) and symptomatic IgA deficiency.

We have assessed the relative severity of the disorder of all family members using the clinical score proposed for the use of subcutaneous immunoglobulin/intravenous immunoglobulin,^21^ which is based on the frequency and severity of immune sequelae of CVID (Table 1). Application of the Ameratunga *et al.*^2, 19^ diagnostic criteria for CVID concluded that the proband (II.2) and her son (III.1) were phenotypically distinct from other family members (clinical score >10) and the highest clinical score was assigned to the proband. Together, these findings indicated that the *TNFRSF13B*/TACI C104R mutation could not be the sole explanation for CVID in this family, prompting a search for other causative genetic mutations.^12^

### Identification of a novel *de novo* mutation of *TCF3* in both severely symptomatic individuals

**Figure 2:**
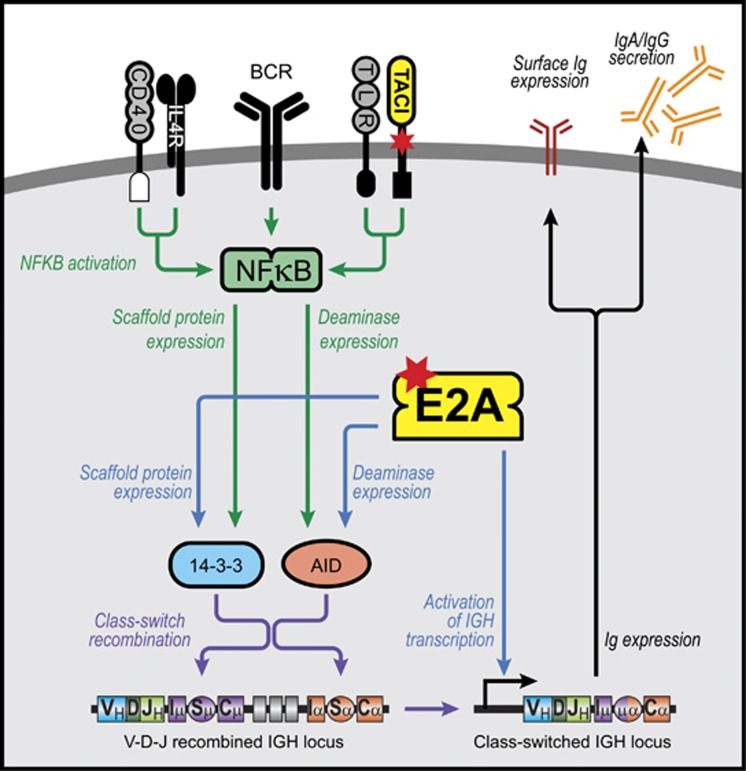
Immunoglobulin isotype switching pathways showing nodes of intracellular signal integration between TACI and *TCF3*/E2A. T-cell-independent isotype switching occurs through TACI and TLRs while T-cell dependent switching occurs through CD40 and IL-4 or IL-21. Ligation of the B-cell receptor synergises with both pathways. *TCF3*/E2A contributes to the expression of AID, 14-3-3γ and Ig production and therefore influences both T-cell-dependent and -independent Ig switching pathways. 14-3-3γ is a scaffolding protein and targets AID to switch regions. Mutations are shown in red stars. BCR—B cell receptor. TLR, Toll-like receptor.

Whole-exome sequencing was performed on II.2, III.1 and III.2 and analysed assuming an autosomal dominant mode of inheritance, where II.2 and III.1 have reference/alternative alleles (REF/ALT) and III.2 is healthy (REF/REF or ALT/ALT). Non-synonymous variants within coding and splice site regions with a minor allelic frequency less than 1% were annotated. Analysis did not reveal evidence of consanguinity and identified 94 rare genetic variants affecting protein sequence that were transmitted by the proband to her son, but not to her daughter (Supplementary Tables 1 and 2). Nine variants affecting genes with known roles in the immune system were genotyped in the entire kindred (Supplementary Table 3). Of these, only a *de novo* frameshift nonsense mutation in *TCF3* encoding the E2A transcription factors E12 and E47^21^ segregated with the two severely symptomatic family members, II.2 and III.1 (Figures 1a and b) in the wider family. *TCF3* plays a critical role in early B-cell development.^22^ It is also thought to play an important role in mature B-cell biology and promotes Ig gene transcription.^23^ Studies have shown that the E2A transcription factors are essential for the expression of several genes involved in the Ig isotype switching and secretion pathway including Activation Induced Deaminase (*AICDA*, which encodes AID) and 14-3-3γ, a scaffolding protein, which targets AID to Ig switch regions (Figure 2).^24^

Insertion of an adenine residue at exon 8 of *TCF3* creates a frameshift leading to a nonsense mutation (T168fsX191, Figure 1b). Threonine at position 168 is the first amino acid to be affected by the frameshift, and results in a stop codon at position 191 (Figure 1c). The presence of the mutation was confirmed by Sanger sequencing and is not expressed in other family members, healthy controls or any publicly available gene databases (Figure 1b). The two severely affected individuals (II.2, III.1) are heterozygous for the mutation, consistent with autosomal dominant inheritance. The mutation was absent in the proband’s parents, indicating its *de novo* origin.

### Haploinsufficiency of E2A in proband (II.2) and her son (III.1)

Neither the mRNA of the mutant *TCF3* T168fsX191 allele nor its truncated protein products (E12 and E47) were expressed, presumably as a result of nonsense mutation mediated decay. The wild-type E2A (E47) protein is poorly expressed in both heterozygous individuals carrying the *TCF3* T168fsX191 mutation (II.2 and III.1), but normal expression was detected in other family members and unrelated healthy controls (Figure 1d). Together these results suggest haploinsufficiency of E2A in affected individuals II.2 and III.1.

### Intracellular NFκB signalling

TACI plays a critical role in immunoglobulin isotype switching particularly when mediated via the T-cell-independent pathway (Figure 2).^25^ TACI acts synergistically with other signalling pathways including Toll-like receptors, the B-cell receptor and CD40 implying a broad range of actions during an immune response.^27^

Both T-cell-dependent and -independent pathways lead to downstream activation of NFκB, expression of activation induced cytidine deaminase (AID) and related molecules (Figure 2). NFκB1 (p105 and its proteolytically cleaved subunit, p50) and NFκB2 (p100 and its active subunit, p52) and their associated transcription factor family members together regulate a large number of target genes that are essential for B-cell development, maturation and differentiation into Ig isotype switched memory and antibody-secreting cells (ASC). We thus first investigated whether NFκB signalling was impaired in the proband to determine the consequences of expressing *TCF3* and *TNFRSF13B*/TACI mutations. Phosphorylation of p105, as well as total p105 and p50 were reduced (~50%) in stimulated peripheral blood mononuclear cells (PBMCs) from the proband (II.2), compared to unrelated healthy controls following stimulation with PMA and ionomycin (Figure 1e). No differences were observed for p100/p52 expression and signalling via the NFκB2 pathway (not shown).

### Immunophenotyping of lymphocyte populations

**Figure 3:**
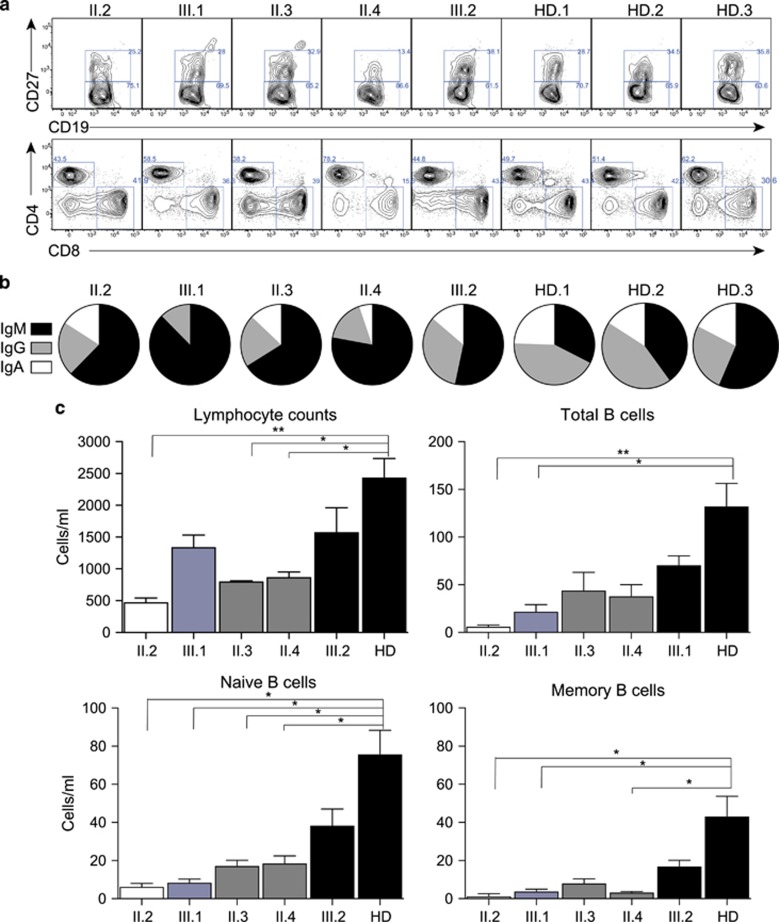
(**a**) Immunophenotyping, proliferation and isotype switching in *TCF3*/ *TNFRSF13B*/TACI mutant B cells. (**a**) Immunophenotyping results indicating proportions of naïve (CD20^+^CD27^−^) and memory (CD20^+^CD27^+^) B cells, and CD4^+^ and CD8^+^ T cells in PBMCs isolated from available family members as indicated, and representative healthy donor controls. (**b**) Relative proportions of IgM/G/A memory B cells from each family member and unrelated healthy donors (each as a proportion of total memory B cells). IgM-expressing cells are shown in black, IgG^−^ in gray and IgA^−^ isotype switched memory B cells in white, as indicated. (**c**) Total numbers of lymphocytes, B cells, naïve (CD20^+^CD27^−^) and memory (CD20^+^CD27^+^) B cells in peripheral blood from each family member and unrelated healthy donors (HD=12). Immunophenotyping and cell counts were performed in two separate experiments.

The two symptomatic individuals (II.2, III.1) bearing the *TCF3* T168fsX191 mutation had a reduced total number of B cells, naïve B cells, as well as a significant reduction in memory B cells, with fewer isotype-switched memory B cells detected (Figures 3a–c). Individuals carrying the *TNFRSF13B*/TACI C104R mutant only (II.3, II.4) also displayed a reduction in the total number of lymphocytes and B cells (Figures 3a and c). No differences in total T-cell number, CD4:CD8 ratios, NK cell or monocytes were observed (Figure 3a and not shown).

### Quantification of a severe *in vitro* antibody production defect by proband naïve B cells demonstrates epistasis

**Figure 4:**
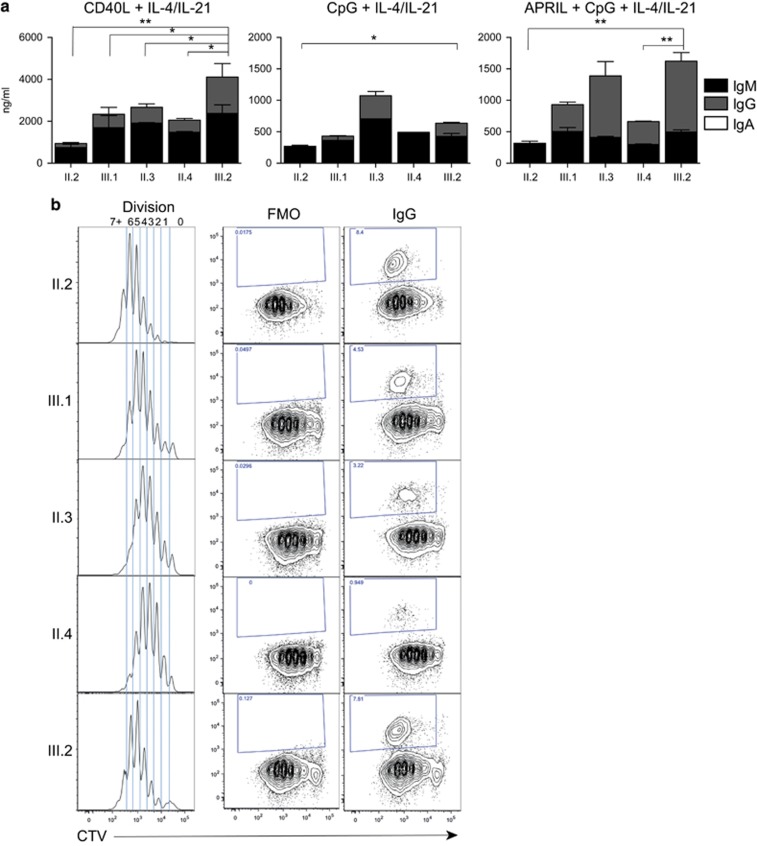
Severe defect in *in vitro* antibody production in proband demonstrates epistasis. (**a**) Immunoglobulin production from supernatants collected from *in vitro* cultures of naïve B cells isolated from PBMCs of each family member, stimulated as indicated with CD40L (100 ng ml^–1^), IL-4 (50 ng ml^–1^), IL-21 (50 ng ml^–1^), CpG (1 μg ml^–1^) and APRIL (500 ng ml^–1^). Supernatants were assessed for secretion of IgG, IgA and IgM as indicated. (**b**) Representative Cell Trace Violet (CTV) plots and IgG isotype switched cells following *in vitro* stimulation of naïve B cells with CD40L+IL-4+IL-21 for 6 days (representative from two independent experiments). Cells were isolated, labelled with CTV, stimulated and collected after 6 days of culture and the division profiles and proportions of IgG expressing cells determined. FMO, fluorochrome minus one.

We next assessed the ability of naïve B cells isolated from each family member to differentiate into ASC leading to the production of Ig following *in vitro* stimulation (Figure 4a). Naïve B cells isolated from the proband (II.2) were able to differentiate and secrete Ig, under both T-cell-dependent (CD40L+IL-4+IL-21) or T-cell-independent (CpG+IL-4+IL-21±APRIL) conditions. However, in each condition, this was consistently less than other family members and was almost exclusively IgM, with very little IgG detected in culture supernatants. The proband’s brother (II.3), who is heterozygous for the *TNFRSF13B*/TACI C104R mutation is able to produce IgG levels comparable to the wild-type family control (III.2, Figure 4a) and unrelated healthy donors (Supplementary Figure 1) via the T-cell-independent pathway. His cells produce lower quantities of IgG through the T-cell-dependent pathway than his niece, (III.2). The TACI/Toll-like receptor pathway can augment T-cell-dependent isotype switching and IgG production,^26^ which may account for the slightly lower IgG levels in comparison with II.3, who is heterozygous for *TNFRSF13B*/TACI C104R mutation, or III.2, who has neither mutation.

Naïve B cells isolated from the brother with the homozygous *TNFRSF13B*/TACI C104R mutation (II.4) were able to produce detectable IgG *in vitro* via the T-cell-independent pathway (Figure 4a). However, his (II.4) cells produced consistently lower levels of IgG compared to his healthy niece (III.2). Previous studies have shown *TNFRSF13B*/TACI C104R homozygous individuals are able to produce some IgG *in vitro* with APRIL stimulation alone.^27^ This is likely to be augmented by Toll-like receptor signalling with CpG as well as IL-4 and IL-21, in our experiments. As expected, his cells produce greater amounts of IgG through his intact T-cell-dependent pathway. The proband’s son (III.1) carrying only the heterozygous *TCF3* T168fsX191 mutation is also able to produce some IgG *in vitro* via activation of both pathways, but at much lower levels than his wild-type sister (III.2). His cells produced higher levels of IgG and IgM than his mother (II.2, who bears both the *TNFRSF13B*/TACI C104R and *TCF3* T168fsX191 mutations).

The combination of *TCF3* T168fsX191and *TNFRSF13B*/TACI C104R mutations in the proband resulted in a greater net effect that the sum of each individual mutation would predict than the sum of deficits observed for each mutation alone (that is, Ig level^III.2^−(Ig^III.2^−Ig^III.1^)+(Ig^III.2^−Ig^II.3^)). When the amount of Ig detected in cultures of *TNFRSF13B*/TACI *TCF3* double mutant naïve B cells following APRIL/CpG stimulation, a much larger deficit is observed compared to TCF3^+/−^ or *TNFRSF13B*/TACI^+/−^ mutant cells alone; that is, the amount of Ig production in the proband (II.2) is much lower than the sum of each individual contribution (by III.1 and II.3). The same is also true for other culture conditions tested (Figure 4a, Supplementary Figure 1). When such a quantitative defect in Ig production is combined with the observed additional defects in total cell number and B-cell development and differentiation, epistatic interaction of *TCF3* and *TNFRSF13B*/TACI mutations is clearly observed in this family.

### Proliferation and isotype switching potential of naïve B cells

We next investigated if the severely reduced *in vitro* antibody production observed in the proband could be explained by impaired proliferation or isotype switching. Naïve B cells were isolated from family members, labelled with the cell division tracking dye, cell trace violet (CTV), and after 6 days of stimulation with CD40L, IL-4 and IL-21, the proliferative and switching potential assessed. Naïve B cells from III.1 (carrying only the *TCF3* T168fsX191 mutation) underwent, on average, slightly fewer rounds of cell division (Mean division number, MDN) than those from his healthy sister (III.2; MDN=3.9 and 4.6, respectively; Figure 4b). A small proliferative difference was also observed in naïve B cells from family members carrying only the *TNFRSF13B*/TACI C104R mutation, either in heterozygous (II.3) or homozygous (II.4) form (MDN=3.3, 3.1, respectively). However, the combination of both mutations in the proband (II.2) showed normal proliferation of naïve B cells, with no direct proliferative defect observed after 6 days of stimulation with CD40L and cytokines (MDN=4.8). Thus, neither mutation prevents B cells from undergoing a relatively healthy proliferative response.

We then examined the ability of stimulated B cells to undergo IgG isotype switching (Figure 4b, right panels). Despite the absence of IgG detected in the supernatants of these cultures, no defect was observed in the generation of isotype switched IgG^+^ cells in II.2 (carrying both *TNFRSF13B*/TACI C104R and *TCF3* T168fsX191 mutations), compared to III.2, who has neither mutation. Her son, III.1, carrying the *TCF3* T168fsX191 mutation only, also generated a similar proportion of IgG^+^ switched cells. However, individuals carrying the *TNFRSF13B*/TACI C104R mutation alone (II.3 and II.4) generated fewer IgG^+^ switched cells from naïve cultures, even in the absence of TACI ligand engagement. Since isotype switching is known to be linked to the number of divisions undergone,^27, 28, 29, 30, 31^ this could be, in part due to the small proliferative delay and reduced number of cells in later divisions observed in TACI-deficient naïve B cells.

### Deficiency of *in vitro* generation of ASC by *TNFRSF13B*/TACI/*TCF3* double mutant naïve B cells

**Figure 5:**
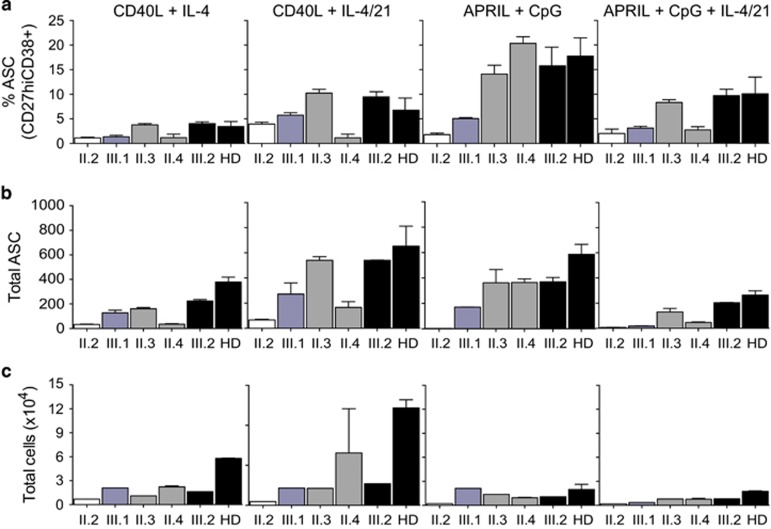
Severe defect in generation of antibody secreting cells in E2A/TACI-deficient cells. (**a**–**c**) Summary graphs from *in vitro* proliferation of naïve B cells stimulated as indicated with CD40L (100 ng ml^−1^), IL-4 (50 ng ml^−1^), IL-21 (50 ng ml^−1^), CpG (1 μg ml^−1^) and APRIL (500 ng ml^−1^) as indicated. Isolated cells were collected after 5 days of culture, cell surface stained and analysed by flow cytometry for the (**a**) proportion of antibody secreting cells (ASC, CD27^hi^CD38^+^) and (**b**) total number of ASC and (**c**) total lymphocyte number. Cell counts and proportion of ASC are shown for the proband, with both *TNFRSF13B*/TACI and *TCF3* mutations in white; her son (III.1), expressing *TCF3* T168fsX191 mutant B cells only (blue); TACI-deficient individuals (II.3, II.4, gray); and wild-type (III.2 and HD, black). Summary graphs of the proportions and total number of differentiated cells for all family members and healthy donors (HD, *n*=4) was performed in two independent experiments.

The above studies showed relatively healthy proliferation and isotype switching by II.2, but markedly reduced secretion of Ig suggesting a defect in development or function of ASC following stimulation.^6, 30^ To investigate a putative differentiation defect in the E2A-TCF3/TACI-deficient B cells, naïve B cells were cultured under T-dependent (CD40L+IL4±21) or T-independent (APRIL+CpG±IL4/21) conditions and the generation of ASC was assessed by flow cytometry after 5 days. The proportion of ASC (CD27^hi^, CD38^+^) generated in cultures from the family wild-type control (III.2) was comparable to that observed in healthy donors, for each stimulation condition (Figure 5). However, a 2–6-fold reduction in the proportion of ASC generated was observed following either T-cell-dependent or T-cell-independent (that is, TACI-dependent) pathways in the proband (II.2) compared with her daughter (III.2) and healthy donor controls (Figure 5a). This effect was even greater (up to 8-fold fewer ASC) when the total number of ASC was calculated following T-dependent stimulation conditions and even more pronounced following TACI-ligand engagement under T-independent conditions (Figure 5b).

Naïve B cells from II.4, homozygous for *TNFRSF13B*/TACI C104R mutation, also showed reduced differentiation into ASC, compared to the healthy family member control (III.2) and unrelated healthy controls, consistent with lower IgG secretion observed in culture supernatants (Figure 4a, Supplementary Figure 1) and the profound hypogammaglobulinemia observed in this patient (Table 1). When total numbers of lymphocytes in these cultures were assessed, fewer cells were present in cultures from all family members (Figure 5c). However, consistently fewer cells were generated in cultures from *TNFRSF13B*/TACI/*TCF3* double mutant B cells from the proband (II.2). Despite normal proportions of divided and isotype-switched IgG^+^ cells in these cultures, a clear B-cell defect was observed in both total cell number and absolute levels of Ig secreted in all mutant naïve B cells, and this defect is most severe in the presence of both mutations, consistent with epistasis.

## Discussion

Epistasis occurs when there are synergistic interactions between two or more genetic loci or their products leading to a phenotype that is either divergent or more severe than the sum of the individual effects.^31^ In humans, epistasis can only be identified when pathogenic mutations are present in two or more genes. Epistasis requires quantification of the consequences of the mutations to demonstrate synergistic effects. The existence and role of epistasis in human disease has been difficult to demonstrate and remains highly controversial since it was first proposed over one hundred years ago.^14, 31, 32^ The predominant difficulty has been the inability to undertake relevant clinical and *in vitro* functional studies to quantify the consequences of multiple genetic mutations.

Previously, the existence of epistasis was inferred from the phenotypic variation in large kindreds carrying mutations of genes responsible for auditory or visual impairment and other congenital disorders.^17^ A recent publication suggested an interaction between *LRBA* and *NEIL3* mutations in a consanguineous Middle Eastern kindred with hypogammaglobulinemia and autoimmunity.^33^ Autosomal recessive LRBA deficiency has been previously described in a number of early-onset CVID-like patients with autoimmune manifestations, including ITP, haemolytic anaemia and inflammatory bowel disease.^34^ The *NEIL3* mutation was present in the three affected children and was also observed in ~2% of individuals of Middle Eastern origin, and thus may be a risk factor for autoimmune disease in this population. No obvious phenotype was observed in an unrelated *NEIL3* homozygous mutant individual. In the affected family, the homozygous *NEIL3* mutation in addition to deleterious mutations in *LRBA* likely contributed to the severe phenotype observed; unfortunately, the three siblings carrying both mutations were deceased, limiting functional studies in this family.

In the kindred presented here, the immune system has offered us an unparalleled opportunity to study epistasis in readily accessible PBMCs.^17^ Individual family members are exemplars for the effects of each mutation or combination on *in vitro* B-cell differentiation, Ig isotype switching and production, which are the ultimate laboratory correlates of late onset antibody failure/immune system failure in CVID. In this family, we have quantified both the clinical severity (using the clinical score) and *in vitro* antibody production to demonstrate a synergistic interaction between the two mutations leading to clinical and genetic epistasis.

**Figure 6:**
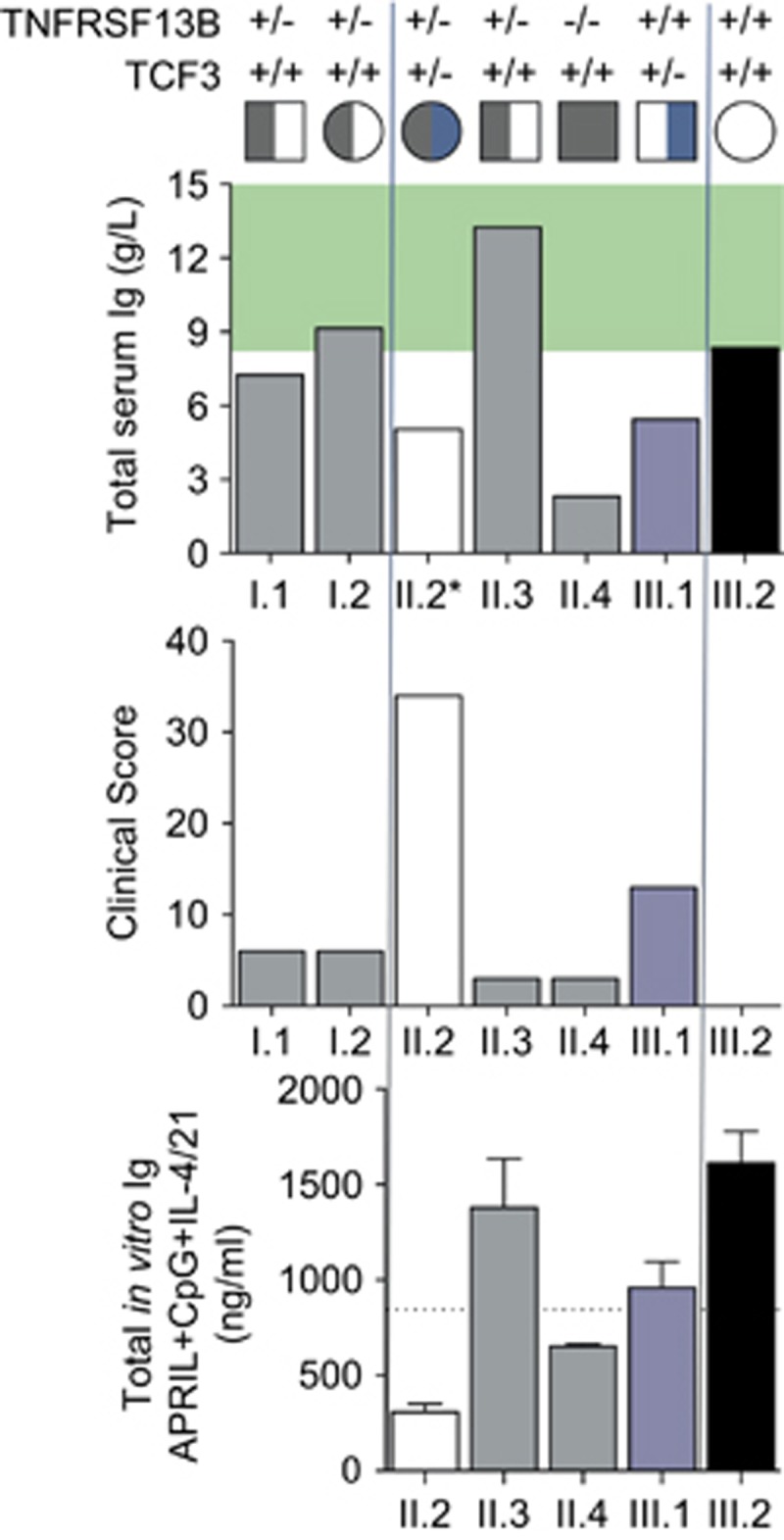
Quantitation of epistatic interactions of *TCF3* and TACI mutations showing a greater net effect than the sum of each individual mutation. Total Serum Ig, clinical score and *TNFRSF13B*/TACI C104R and *TCF3* T161fsX191 genotype for each family member, as indicated. The serum IgG for the proband II.2 was obtained in 2002. Normal serum Ig ranges (g l^−1^) shaded in green. Lower graph: summary of total Ig levels detected in naïve B-cell cultures for each available family member, stimulated with APRIL, CpG, IL-4+IL-21 for 6 days as described. Indicated line is the total Ig level expected for the proband (II.2) calculated from the sum of deficits observed for each mutation alone (that is Ig level^III.2^− (Ig^III.2^−Ig^III.1^)+(Ig^III.2^−Ig^II.3^)). Note that the clinical score for individual III.2 is 0.

Both the TACI and *TCF3*/E2A networks share nodes of intracellular signal integration (Figure 2), mutations of which appear to have synergistically (epistatically) impaired B-cell function in the proband (II.2). In her case, the two mutations, which lie in tandem along the Ig isotype switching and secretion pathway (Figure 2), lead to severely impaired B-cell differentiation and production of IgG *in vitro* (Figures 3 and 4) and in severe clinical disease (Table 1, summarised in Figure 6). The proband, carrying both mutations shows the largest defect *in vitro* after isolated naïve B cells are specifically engaged via CD40, APRIL or Toll-like receptorss and is much more severely affected than her parents, her son and her siblings. Her *in vitro* IgG production is substantially lower than that of her *TNFRSF13B*/TACI C104R heterozygous brother (II.3) and her *TCF3* T168fsX191 heterozygous son (III.1), who individually bear each mutation. While clear defects in B-cell development, isotype switching and differentiation into ASCs were observed in both individuals (II.2 and III.1) carrying the mutant *TCF3* allele, the additional effect of the C104R TACI mutation in the proband (II.2) resulted in a more severe B lymphocyte cellular phenotype, consistent with epistasis.

Here, immunophenotyping of lymphocyte populations and *in vitro* assessment of differentiation into isotype switched memory B cells did not reveal such a severe block in B-cell development. Instead, we observed a marked reduction in the total number, but not the proportion, of isotype switched and total memory B cells present in the proband, who carries both *TCF3* and TACI gene mutations, as well as her son, who carries only the *TCF3* mutation and her brother, homozygous for the TACI gene variant only. These data suggest that neither mutation is intrinsically necessary for the generation of memory B cells or for Ig isotype switching, but may be critical for the survival and/or maintenance of the populations. Further investigations will be necessary to determine the relative contributions of these mutations on memory B-cell persistence.

The quantification of the phenotypic disease severity also mirrors the pattern of mutations of these two unrelated genes (Table 1,Figure 6). The proband, the only family member to carry both mutations, is much more severely affected than her parents, her son and her siblings. Her clinical score is much higher than the sum of her son (III.1) and any of her *TNFRSF13B*/TACI C104R heterozygous family members (I.1, I.2, II.3; Figure 6), which is consistent with epistasis.^13^ It should be noted that the proband’s serum IgG level was measured over 15 years ago, prior to Ig recommencement; therefore, no assumptions can be made about her current levels. Furthermore, if the total level of Ig secreted in these cultures is compared for each family member, then the net deficit for the proband carrying both mutations is much greater than sum of each individual deficit. A comparison of the amount of Ig detected in cultures of *TCF3*/TACI double mutant naïve B cells following APRIL/CpG stimulation reveals a larger deficit than the Ig observed in *TCF3*^+/−^ or TACI^+/−^ mutant cells alone; that is, the amount of Ig production in the proband (II.2) is lower than the sum of each individual contribution (by III.1 and II.3, Figure 6). When such a defect in Ig production is combined with the observed additional defects in total cell number and possibly B-cell development, epistatic interactions of TCF3 and TACI mutations is clearly observed in this family.

The novel *TCF3* T168fsX191 mutation presented here has a clear pathogenic effect on total B cell and switched memory B-cell development, generation of ASC and Ig production.

The mutation has arisen *de novo* in the proband, co-segregates with the disease phenotype, and is absent in over 60 000 individuals without overt immunodeficiency phenotypes corresponding to a minor allele frequency less than 10^−5^ (Exome Aggregation Consortium).

There is also convincing evidence from other human and animal studies that the *TCF3* T168fsX191mutation is pathogenic in this family. In another recent study, four unrelated individuals with *de novo* heterozygous E55K missense mutations of *TCF3* presented with a severe B-cell defect and agammaglobulinemia, and here a dominant negative mechanism was suggested.^35^

Our data suggest the *TCF3* T168fsX191 mutation is more likely to cause its effects through haploinsufficiency in this kindred, leading to a distinct phenotypic presentation. Another recent publication suggested an association of sequence variations in *TCF3* in a patient with CVID,^36^ although detailed functional studies were not presented. In addition to inherited disease, a recent report of monozygotic twins discordant for CVID, demonstrated differential methylation signatures of *TCF3* between the unaffected and affected twins. The authors postulated impaired activity of *TCF3*/E2A accounted for the presence of disease.^37^

Two independent studies of gene-targeted mice with *TCF3* haploinsufficiency have shown reduced numbers of B cells and impaired lymphoid cell development.^23, 38^ Similarly, reduced expression of *TCF3*/E2A has been implicated in equine CVID.^39^ There is thus strong support from human, murine and equine studies for the pathogenicity of the *TCF3* T168fsX191 mutation in our family.

Our study also offers new insights into the role of *TNFRSF13B*/TACI mutations in the pathogenesis of CVID.^11^ The C104R mutant is a low frequency variant in population databases (0.32% in Exome Aggregation Consortium) and although earlier publications considered this variant to be disease-causing and expressed in up to 10% of CVID patient cohorts,^40^ it, and other *TNFRSF13B*/TACI variants were subsequently found to be present in ~2% of healthy control populations.^41^ Although functional studies of C104R mutant alleles have demonstrable defects in B-cell development, switching and differentiation, it is considered a risk allele for CVID, with a relative risk of 4.2^41^ and it has long been speculated that second mutations may be identified in these families.^13^ This study is the first demonstration of such digenic inheritance in a CVID-like disorder.

In this family, the *TNFRSF13B*/TACI C104R mutation appears to demonstrate a gene dosage effect on serum IgG levels. The brother who is homozygous (II.4) for the *TNFRSF13B*/TACI C104R mutation has the lowest IgG levels, and consistently generated fewer isotype switched and differentiated ASC *in vitro*, compared with other family members who are heterozygotes.^20^ The presence of concomitant mutations, such as the *TCF3* T168fsX191 mutation seen in the proband, may explain the variable penetrance and expressivity of *TNFRSF13B*/TACI mutations in CVID.

Individuals with digenic disorders will pose challenges for preimplantation genetic diagnosis and chorionic villus sampling. Here, we have demonstrated that the *TCF3* T168fsX191 mutation has a more detrimental effect on the phenotype in this pedigree. It could be argued that the *TNFRSF13B*/TACI C104R mutation has a modifying effect on the phenotype and is relatively benign in this family. Hence, priority should be given to identifying the *TCF3* T168fsX191 mutation for preimplantation genetic diagnosis and/or chorionic villus sampling.

Based on both clinical and laboratory quantification, it appears neither the *TNFRSF13B*/TACI C104R mutation nor the *TCF3* T168fsX191 mutation alone is sufficient to cause the complete, severe CVID-like disorder and SLE observed in the proband. This is the first example of late onset antibody failure/immune system failure resulting from epistatic interactions of two independent monogenic defects leading to a CVID-like disorder.^17^ We anticipate future genomic sequencing and functional validation studies will reveal additional instances of polygenic pathogenic mutations and epistatic gene interactions in other families. Classification of such primary immunodeficiency disorder patients will require a new category for multigenic disorders. This family fulfils Bateson’s astute predictions of human epistasis made over a century ago.^13^

## Methods

### Study participants/human samples

Peripheral blood mononuclear cells (PBMCs) were isolated from healthy control subjects (Volunteer Blood Donor Registry and Auckland City Hospital) and from the proband and family members, following informed consent. All studies were approved by ADHB (3435), NZ Ministry of Health (MEC/06/10/134) and Walter and Eliza Hall Institute (WEHI) Human Research Ethics Committee (HREC 10/02).

### Whole exome sequencing

We undertook whole exome sequencing on individuals as indicated (Figure 1a). Rare variants (frequency <0.01 in the Exome Aggregation Consortium,^42^ 1000 Genomes, and HapMap projects, or our in-House database) likely affecting protein primary sequence and co-segregating with CVID-like symptoms (present in II.2 and III.2 but absent in II.1 and III.2) were shortlisted for interpretation of disease causality.

### Sanger sequencing

All PCR amplifications were performed as described (Roche protocol for Faststart Taq DNA polymerase). The following PCR primers were used (i) *TNFRSF13B* sense: 5′-TACTTGGCTTACTCTGGAAT-3′ and anti-sense: 5′-CATTTGCTTGGACTCTGG-3′ and (ii) *TCF3* sense: 5′-TCTCTTGACCTCGTGATCTG-3′, anti-sense 5′-GACTCACCGAGGATGGAA-3′.

DNA sequencing was performed with Big Dye Terminator cycle sequencing on an ABI 3130 × l Genetic Analyzer according to the manufacturer’s standard protocol and reagents (Applied Biosystems, Waltham, MA, USA). Sequence electropherograms were compared with wild-type sequences using SeqMan v5.01 software (DNASTAR, Madison, WI, USA).

### Lymphocyte phenotyping and naïve B-cell isolation

PBMCs were isolated from whole blood collected from family members and healthy donors by ficoll-histopaque gradient centrifugation. For phenotypic staining, the following monoclonal antibodies were used: CD3-APC-H7, CD4-PerCP-Cy5.5, CD38-PerCP-Cy5.5, CD10-PECF594, CD21-APC, IgG PeCy7, CD14-PerCP, CD123-PE, CD56-PeCy7, CD11c-APC, CD16-APC-H7 (BD Biosciences, San Diego, CA, USA), CD8-APC-EF780, CD27-APC-EF780 (eBioscience, San Diego, CA, USA), CD19-BV650, CD24-BV605 (Biolegend, San Diego, CA, USA) and IgA-PE (Miltenyi Biotech, Bergisch Gladbach, Germany). Naïve B cells were enriched by negative selection using B-cell isolation kit (Stemcell, Vancouver, BC, Canada). Naïve B-cell purity was verified by flow cytometry to 98% purity.

### Cell stimulation protocols

Purified naïve B cells were cultured in B-cell medium (RPMI 1640 containing L-glutamine; Invitrogen Life Technologies, CA, USA), supplemented with 10% fetal calf serum (FCS) (Invitrogen Life Technologies, Waltham, MA, USA), 10 mM 4-(2-hydroxyethyl)-1-piperazineethansulfonic acid (HEPES) (pH 7.4; Sigma-Aldrich, St Louis, MO, USA), 0.1 mM nonessential amino acid solution (Sigma-Aldrich), 1 mM sodium pyruvate (Invitrogen Life Technologies), 60 mg ml^−1^ penicillin, 100 mg ml^−1^ streptomycin, 40 mg ml^−1^ transferrin (Sigma-Aldrich), and 20 μg ml^−1^ Normocin (InVivogen, San Diego, CA, USA); and stimulated with 100 ng ml^−1^ CD40L alone (Enzo, Farmingdale, NY, USA) or with IL-4 (100 ng ml^−1^), IL-21 (50 ng ml^−1^; both Peprotech), or CpG 2006 (1 μg ml^−1^, Invitrogen, Carlsbad, CA, USA), APRIL (500 ng ml^−1^, Adipogen, San Diego, CA, USA), in the presence or absence of IL-4 and IL-21. For some experiments, B cells were labelled with division-tracking dye cell trace violet (CTV, Invitrogen).^32^ For phenotypic and functional analysis, cells were cultured in 96-well plates for 5 or 6 days, collected, stained with CD20, CD27, CD38, IgG, IgM, IgA and the proportion of isotype switched and differentiated antibody secreting cells determined as previously described.^6^ Secreted IgM, IgG and IgA levels were determined by Ig Heavy chain-specific immunoassays as previously described.^6^

### Western blotting

Freshly isolated PBMCs were either analysed at rest or following stimulation with PMA/ionomycin (50 ng ml^−1^ and 500 ng ml^−1^, respectively, Sigma). Resting or stimulated PBMCs were washed twice with ice-cold phosphate buffered saline (PBS), and lysed in sodium dodecyl sulfate (SDS) lysis buffer according to the manufacturer’s protocol (Cell Signaling Technology, Danvers, MA, USA). Cell lysates were separated by SDS-PAGE gel electrophoresis, and transferred to polyvinylidene difluoride (PVDF) membranes (Millipore, Billerica, MA, USA). Membranes were immunoblotted with antibodies (Cell Signaling, Danvers, MA, USA) against E2A (D2B1), phospho-p105 (Ser933; 18E6), phospho-p100 (Ser866/870), p105/p50 (#3035), p100/p52 (18D10), and actin (Millipore). NFκB bound antibodies were visualised using SuperSignal West Pico (Thermo Scientific, Waltham, MA, USA) or ECL plus (GE Healthcare, Chicago, IL, USA) and the chemiluminescence detection system by Fujifilm Las-3000. Staining for actin was used as a control for protein loading.

## Figures and Tables

**Table 1:** Clinical characteristics of the kindred

*Individual*	*Age/sex*	*TACI (TNFRSF13B) mutation*	*TCF3 mutation*	*IgG (g l*^*−1*^)	*IgA (g l*^*−1*^)	*IgM (g l*^*−1*^)	*Memory B cells*	*Vaccine responses*	*Autoimmunity*	*Infectious history*	*Classification*^a^	*Clinical score*^b^
I.1	88/M	C104R	Nil	6	0.9	0.4	ND	ND	ITP	Respiratory	sHGUS	6
I.2	93/F	C104R	Nil	6.3	0.9	2.0	ND	ND	ITP	Respiratory	sHGUS	6
II.2	61/F	C104R	T168fsX191	4.5^c^	0.2	0.4	Reduced switched memory	Impaired Pneumovax, diphtheria, tetanus toxoids	SLE Hashimoto’s Thyroditis	Respiratory Meningitis Chronic sinusitis Gastrointestinal	CVID-like	34
II.3	55/M	C104R	Nil	9.7	1.5	2.1	Normal	ND	Mild cytopenia	Well	Well	3
II.4	58/M	C104R/C104R	Nil	1.6	0.11	0.67	Reduced switched memory	Transient	Mild cytopenia	Well	Well	3
III.1	35/M	Nil	T168fsX191	5.5	<0.07	0.4	Reduced switched memory	Impaired Pneumovax, diphtheria, tetanus toxoids	T1D Antiparietal Synovitis	Sinusitis Chronic tonsillitis	‘sHGUS’ IgAd	13
III.2	33/F	Nil	Nil	7.1	0.8	0.5	Normal	ND	Nil	Well	Well	0

Abbreviations: sHGUS, symptomatic hypogammaglobulinemia of uncertain significance; SLE, Systemic Lupus Erythematosus; CVID, Common Variable Immunodeficiency Disorders; IVIG, intravenous immunoglobulin; SCIG, subcutaneous immunoglobulin.

a Classification according the Ameratunga *et al.*^2^ criteria. sHGUS- symptomatic hypogammaglobulinemia of uncertain significance, IgAd, IgA deficiency; F denotes females, M denotes males. III.1 has been re-designated ‘sHGUS’, as he has an underlying genetic defect. II.2 has been classified as CVID-like, as she now has an underlying genetic cause. Reference ranges IgG 7–14 g l^−1^, IgA 0.8–4.0 g l^−1^, IgM 0.4–2.5 g l^−1^.

b We have used the clinical score as an index of severity based on sequelae of the disorder.^21^

c IgG level of the proband II.2 was obtained in 2002, during a break from IVIG treatment, as result of adverse reactions; her current IgG is unknown because of SCIG treatment. ND, not done; T1D, type 1 diabetes.
