# Supplementary figures and images for: Epistatic interactions between mutations of TACI (TNFRSF13B) and TCF3 result in a severe primary immunodeficiency disorder and systemic lupus erythematosus
Source: Clin Transl Immunology. 2017 Oct 20;6(10):e159–. doi: 10.1038/cti.2017.41 (PMC5671988; doi:10.1038/cti.2017.41)

SUPPLEMENTARY FIGURE 1

A

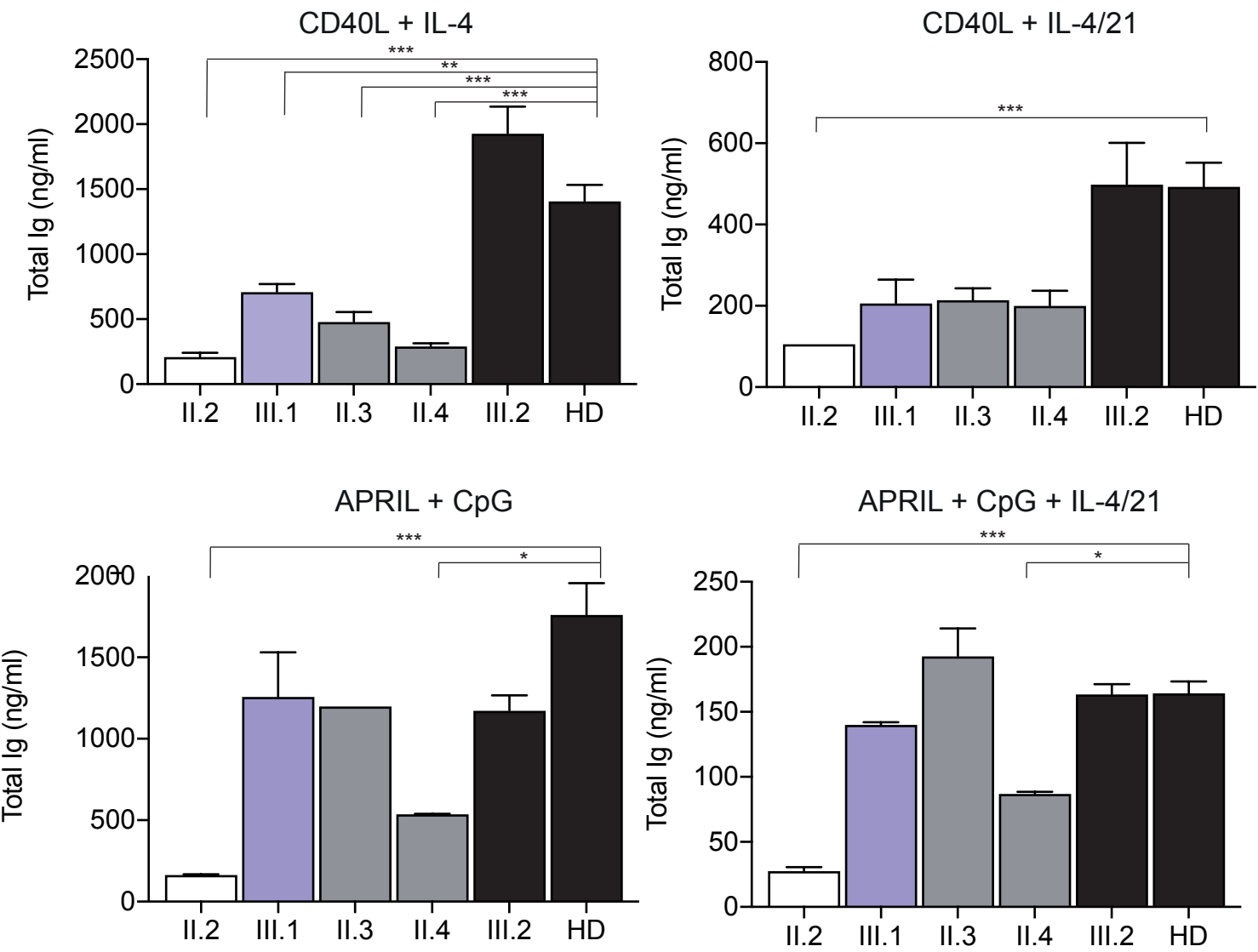

Supplement: Supplementary Figure 1 [file cti201741x1.pdf]
