# Supplementary material for: Epistatic interactions between mutations of TACI (TNFRSF13B) and TCF3 result in a severe primary immunodeficiency disorder and systemic lupus erythematosus
Source: Clin Transl Immunology. 2017 Oct 20;6(10):e159–. doi: 10.1038/cti.2017.41 (PMC5671988; doi:10.1038/cti.2017.41)
Supplement: Supplementary Table 1 [file cti201741x2.pdf]

**Supplementary Table 1: Variant filtration**

| | | | | | |
|---|---|---|---|---|---|
| High-confidence variants discovered in family | | | 105,815 (63,994 on target) | | |
| Variant genotype shared II.2 and III.3 | | | 62,798 (37,977) | | |
| Variant ALT allele <0.01 in global 'healthy' populations and rare in-house | | | 850 (524) | | |
| Variant likely to affect protein sequence | | | 142 (139) | | |
| Genotype not shared with II.1 or III.2 | | | 94 (91) | | |
| Interpreted for causality under genetic model | DOM | CR+DOM | CR | HOM REC | DE NOVO |
| | 77 | 8 | 9 | 0 | 0 |
| Variants selected for family-wide genotyping | 9 | 0 | 0 | 0 | 0 |
| Variants co-segregating with disease severity | 1 | 0 | 0 | 0 | 0 |

| | |
|---|---|
| DOM | variant interpreted under dominant genetic model |
| CR+DOM | variant interpreted under compound recessive and dominant genetic models |
| CR | variant interpreted under compound recessive model |
| HOM REC | variant interpreted under homozygous and hemizygous genetic models |
| DE NOVO | variant interpreted under *de novo* genetic model |
| on-target | site is directly targeted by the exome enrichment kit |
