# Supplementary material for: Epistatic interactions between mutations of TACI (TNFRSF13B) and TCF3 result in a severe primary immunodeficiency disorder and systemic lupus erythematosus
Source: Clin Transl Immunology. 2017 Oct 20;6(10):e159–. doi: 10.1038/cti.2017.41 (PMC5671988; doi:10.1038/cti.2017.41)
Supplement: Supplementary Table 2 [file cti201741x3.pdf]

Supplementary Table 2: Characteristics of filtered variants

Chr	Pos (GRCh37.p13)	rs identifier	REF	ALT	II.1	II.2	III.1	III.2	Gene	Effect	HGVS classification	interpreted under genetic model	Selected for kindred-wide genotyping
1	32,098,120	rs144874149	T	C	0/0	0/1	0/1	0/0	PEF1	NON_SYNONYMOUS_CODING	p.Ile201Val/c.601T>C	dominant	
1	38,019,657	rs200160575	G	T	0/0	0/1	0/1	0/0	SNIP1	NON_SYNONYMOUS_CODING	p.Ser58Arg/c.174G>T	dominant	
1	45,295,296	rs139624405	C	T	0/0	0/1	0/1	0/0	PTCH2	NON_SYNONYMOUS_CODING	p.Arg358His/c.1073C>T	dominant	
1	152,081,632	rs2496251	C	G	0/0	0/1	0/1	0/0	TCHH	NON_SYNONYMOUS_CODING	p.Arg1354Pro/c.4061C>G	dominant	
1	153,907,306	.	T	TGCTGC,TGC	0/0	2/2	0/2	0/2	DENND4B	FRAME_SHIFT	p.X901X/c.2702*>+GCTGC	compound recessive	
1	160,011,262	rs142596580	T	C	0/0	0/1	0/1	0/0	KCNJ10	NON_SYNONYMOUS_CODING	p.Lys354Arg/c.1061T>C	dominant	
1	169,825,081	rs140724273	C	T	0/0	0/1	0/1	0/0	SCYL3	NON_SYNONYMOUS_CODING	p.Val444Ile/c.1330C>T	dominant	
1	203,316,842	.	T	C	0/0	0/1	0/1	0/0	FMOD	NON_SYNONYMOUS_CODING	p.Asn186Ser/c.557T>C	dominant	
1	205,649,537	rs189035913	G	A	0/0	0/1	0/1	0/0	SLC45A3	START_GAINED		dominant	
1	227,307,541	.	G	C	0/0	0/1	0/1	0/0	CDC42BPA	NON_SYNONYMOUS_CODING	p.Ile537Met/c.1611G>C	dominant	
1	236,988,671	rs201871910	C	T	0/0	0/1	0/1	0/0	MTR	NON_SYNONYMOUS_CODING	p.Thr300Met/c.899C>T	dominant	
2	63,272,617	.	G	C	0/0	0/1	0/1	0/0	EHBP1	NON_SYNONYMOUS_CODING	p.Glu1155Asp/c.3465G>C	dominant	
2	107,040,309	.	C	G	0/0	0/1	0/1	0/0	RGPD3	NON_SYNONYMOUS_CODING	p.Gly1372Arg/c.4114C>G	dominant	YES
2	131,116,531	rs150560639	A	G	0/0	0/1	0/1	0/0	PTPN18	NON_SYNONYMOUS_CODING	p.Tyr62Cys/c.185A>G	dominant	
2	211,168,277	rs181132123	G	A	0/0	0/1	0/1	0/0	MYL1	START_GAINED		dominant	
2	242,743,532	rs150107870	T	C	0/0	0/1	0/1	0/0	GAL3ST2	NON_SYNONYMOUS_CODING	p.Leu383Pro/c.1148T>C	dominant	
3	12,858,380	rs201105764	T	A	0/0	0/1	0/1	0/1	CAND2	NON_SYNONYMOUS_CODING	p.Leu557Gln/c.1670T>A	compound recessive	
3	129,290,644	rs150425885	G	C	0/0	0/1	0/1	0/0	PLXND1	NON_SYNONYMOUS_CODING	p.Leu1041Val/c.3121G>C	dominant	YES
4	7,705,999	rs201433688	C	T	0/0	0/1	0/1	0/1	SORCS2	NON_SYNONYMOUS_CODING	p.Thr447Met/c.1340C>T	compound recessive	
4	7,726,982	rs201083224	G	A	0/0	0/1	0/1	0/1	SORCS2	NON_SYNONYMOUS_CODING	p.Val733Ile/c.2197G>A	compound recessive	
4	106,111,551	.	T	C	0/0	0/1	0/1	0/0	TET2	START_GAINED		dominant	YES
4	121,961,159	rs201745396	G	A	0/0	0/1	0/1	0/0	NDNF	NON_SYNONYMOUS_CODING	p.Thr80Met/c.239G>A	dominant	
5	1,225,698	.	G	T	0/0	0/1	0/1	0/0	SLC6A18	NON_SYNONYMOUS_CODING	p.Val36Leu/c.106G>T	dominant	
5	5,463,856	.	C	T	0/0	0/1	0/1	0/0	ICE1	NON_SYNONYMOUS_CODING	p.Ser1470Phe/c.4409C>T	dominant	YES
5	11,384,801	.	G	C	0/0	0/1	0/1	0/0	CTNND2	NON_SYNONYMOUS_CODING	p.Leu294Val/c.880G>C	dominant	
5	148,406,624	.	C	T	0/0	0/1	0/1	0/0	SH3TC2	NON_SYNONYMOUS_CODING	p.Ala438Thr/c.1312C>T	dominant	
6	136,882,715	rs41288957	C	T	0/0	0/1	0/1	0/1	MAP3K5	NON_SYNONYMOUS_CODING	p.Asp1315Asn/c.3943C>T	compound recessive	
6	159,104,008	rs139839252	A	G	0/0	0/1	0/1	0/0	SYTL3	NON_SYNONYMOUS_CODING	p.Asn7Ser/c.20A>G	dominant	
7	47,925,535	rs76044743	G	A	0/0	0/1	0/1	0/0	PKD1L1	NON_SYNONYMOUS_CODING	p.Thr985Ile/c.2954G>A	compound recessive and dominant	
7	73,249,125	rs139220872	G	A	0/0	0/1	0/1	0/0	WBSCR27	NON_SYNONYMOUS_CODING	p.Pro229Leu/c.686G>A	dominant	
7	100,420,010	rs147563837	G	A	0/0	0/1	0/1	0/0	EPHB4	NON_SYNONYMOUS_CODING	p.Pro231Ser/c.691G>A	dominant	
7	150,034,533	rs61744057	G	A	0/0	0/1	0/1	0/0	LRRC61	NON_SYNONYMOUS_CODING	p.Glu195Lys/c.583G>A	dominant	
8	103,291,407	rs35794419	A	G	0/0	0/1	0/1	0/0	UBR5	NON_SYNONYMOUS_CODING	p.Ser2005Pro/c.6013A>G	dominant	
9	4,117,933	rs72687988	C	G	0/0	0/1	0/1	0/0	GLIS3	NON_SYNONYMOUS_CODING	p.Glu360Asp/c.1080C>G	dominant	
9	6,981,115	rs35049841	G	A	0/0	0/1	0/1	0/0	KDM4C	NON_SYNONYMOUS_CODING	p.Arg190Gln/c.569G>A	dominant	
9	134,025,787	.	T	C	0/0	0/1	0/1	0/0	NUP214	NON_SYNONYMOUS_CODING	p.Ile696Thr/c.2087T>C	dominant	
9	134,322,541	.	C	T	0/0	0/1	0/1	0/0	PRRC2B	NON_SYNONYMOUS_CODING	p.Arg309Cys/c.925C>T	dominant	
9	139,837,138	rs148935719	G	A	0/0	0/1	0/1	0/0	FBXW5	NON_SYNONYMOUS_CODING	p.Ala179Val/c.536G>A	dominant	
10	79,584,179	rs146507058	C	T	0/0	0/1	0/1	0/0	DLG5	NON_SYNONYMOUS_CODING	p.Arg782His/c.2345C>T	dominant	
10	127,426,981	.	C	G	0/0	0/1	0/1	0/0	C10orf137	NON_SYNONYMOUS_CODING	p.Leu616Val/c.1846C>G	dominant	
11	6,580,282	rs199875777	C	T	0/0	0/1	0/1	0/0	DNHD1	NON_SYNONYMOUS_CODING	p.Thr3121Ile/c.9362C>T	dominant	
11	8,642,003	rs150122555	T	C	0/0	0/1	0/1	0/0	TRIM66	NON_SYNONYMOUS_CODING	p.Tyr1045Cys/c.3134T>C	compound recessive and dominant	
11	18,743,511	rs200152516	C	T	0/0	0/1	0/1	0/0	IGSF22	NON_SYNONYMOUS_CODING	p.Gly63Ser/c.187C>T	dominant	
12	48,723,318	.	G	C	0/0	0/1	0/1	0/0	H1FNT	NON_SYNONYMOUS_CODING	p.Glu82Gln/c.244G>C	compound recessive and dominant	
12	71,016,226	rs111592755	T	C	0/0	0/1	0/1	0/0	PTPRB	NON_SYNONYMOUS_CODING	p.Thr217Ala/c.649T>C	compound recessive and dominant	
12	72,070,803	rs142758041	A	G	0/0	0/1	0/1	0/0	THAP2	NON_SYNONYMOUS_CODING	p.Glu201Gly/c.602A>G	dominant	
12	92,818,706	rs201506596	T	C	0/0	0/1	0/1	0/0	CLLU1	NON_SYNONYMOUS_CODING	p.Tyr84His/c.250T>C	dominant	YES
12	94,613,883	rs114758530	A	G	0/0	0/1	0/1	0/0	PLXNC1	NON_SYNONYMOUS_CODING	p.Lys549Arg/c.1646A>G	dominant	YES
12	100,928,724	rs189042762	C	T	0/0	0/1	0/1	0/0	NR1H4	NON_SYNONYMOUS_CODING	p.His168Tyr/c.502C>T	dominant	
12	121,458,612	.	C	T	0/0	0/1	0/1	0/0	OASL	NON_SYNONYMOUS_CODING	p.Glu433Lys/c.1297C>T	dominant	YES
14	45,478,336	.	G	A	0/0	0/1	0/1	0/0	FAM179B	NON_SYNONYMOUS_CODING	p.Gly1042Glu/c.3125G>A	dominant	

14	63,416,806	rs41285522	A	G	0/0	0/1	0/1	0/0	KCNH5	NON_SYNONYMOUS_CODING	p.Phe414Leu/c.1240A>G	dominant	
14	102,027,868	rs199500701	G	A	0/0	0/1	0/1	0/0	DIO3	NON_SYNONYMOUS_CODING	p.Gly12Glu/c.35G>A	dominant	
15	62,212,770	rs139993005	T	C	0/0	0/1	0/1	0/0	VPS13C	NON_SYNONYMOUS_CODING	p.His2337Arg/c.7010T>C	dominant	
15	77,363,263	rs111428129	C	T	0/0	0/1	0/1	0/0	TSPAN3	NON_SYNONYMOUS_CODING	p.Val12Met/c.34C>T	dominant	
15	79,296,192	rs113161554	C	T	0/0	0/1	0/1	0/0	RASGRF1	NON_SYNONYMOUS_CODING	p.Asp33Asn/c.97C>T	dominant	
15	90,774,333	.	G	C	0/0	0/1	0/1	0/0	CIB1	NON_SYNONYMOUS_CODING	p.Ile153Met/c.459G>C	dominant	
15	91,537,729	.	G	A	0/0	0/1	0/1	0/0	PRC1	START_GAINED		dominant	
17	40,172,150	.	T	G	0/0	0/1	0/1	0/0	NKIRAS2	START_GAINED		dominant	
17	40,452,271	.	G	C	0/1	0/1	0/1	0/1	STAT5A	NON_SYNONYMOUS_CODING	p.Glu133Gln/c.397G>C	compound recessive	
17	41,116,127	rs140103494	C	T	0/0	0/1	0/1	0/0	PTGES3L-AARSD1	NON_SYNONYMOUS_CODING	p.Gly139Glu/c.416C>T	dominant	
17	61,972,766	rs9909776	A	G	0/0	0/1	0/1	0/1	CSH1	NON_SYNONYMOUS_CODING	p.Tyr175His/c.523A>G	compound recessive	
17	61,972,786	rs61763998	G	T	0/0	0/1	0/1	0/1	CSH1	STOP_GAINED	p.Ser168*/c.503G>T	compound recessive	
17	72,889,676	.	G	GGGCTCCGT	1/2	1/1	1/1	1/2	FADS6	CODON_CHANGE_PLUS_CODON_INSERTI	p.Pro6X/c.17*>+GGCTCCGTAGGTT	dominant	
18	44,584,608	.	T	G	0/0	0/1	0/1	0/0	KATNAL2	NON_SYNONYMOUS_CODING	p.Met112Arg/c.335T>G	dominant	
18	45,566,448	rs61738460	C	T	0/0	0/1	0/1	0/0	ZBTB7C	NON_SYNONYMOUS_CODING	p.Gly344Glu/c.1031C>T	dominant	
19	1,623,996	.	G	GT	0/0	0/1	0/1	0/0	TCF3	FRAME_SHIFT	p.Thr168fsX191/c.349*>+T	dominant	YES
19	1,827,020	.	TGGA	T,TGGAGGA	0/1	0/2	0/2	0/0	REXO1	CODON_CHANGE_PLUS_CODON_INSERTI	p.Ser588X/c.1764*>+GGA	dominant	
19	1,923,176	rs116369903	C	T	0/0	0/1	0/1	0/0	SCAMP4	NON_SYNONYMOUS_CODING	p.Ala134Val/c.401C>T	dominant	
19	6,832,126	rs144739934	G	A	0/0	0/1	0/1	0/0	VAV1	NON_SYNONYMOUS_CODING	p.Glu378Lys/c.1132G>A	dominant	
19	16,799,084	.	G	A	0/0	0/1	0/1	0/1	TMEM38A	NON_SYNONYMOUS_CODING	p.Gly268Ser/c.802G>A	compound recessive	
19	35,422,772	rs146842588	G	A	0/0	0/1	0/1	0/0	ZNF30	NON_SYNONYMOUS_CODING	p.Gly12Glu/c.35G>A	dominant	
19	36,297,671	rs148996461	G	A	0/0	0/1	0/1	0/0	PRODH2	NON_SYNONYMOUS_CODING	p.Ala323Val/c.968G>A	dominant	
19	36,339,016	rs146290709	C	T	0/0	0/1	0/1	0/0	NPHS1	NON_SYNONYMOUS_CODING	p.Arg456Gln/c.1367C>T	dominant	
19	38,965,975	rs137933390	A	G	0/0	0/1	0/1	0/0	RYR1	NON_SYNONYMOUS_CODING	p.Lys1393Arg/c.4178A>G	dominant	
19	40,875,860	.	G	A	0/0	0/1	0/1	0/0	PLD3	NON_SYNONYMOUS_CODING	p.Val159Met/c.475G>A	dominant	
19	43,373,084	rs149977860	T	G	0/0	0/1	0/1	0/0	PSG1	NON_SYNONYMOUS_CODING	p.Tyr178Ser/c.533T>G	dominant	
19	43,411,250	.	G	A	0/0	0/1	0/1	0/0	PSG6	NON_SYNONYMOUS_CODING	p.Ala262Val/c.785G>A	dominant	
19	43,690,539	rs140275565	G	T	0/0	0/1	0/1	0/0	PSG5	NON_SYNONYMOUS_CODING	p.Pro7Thr/c.19G>T	dominant	
19	50,368,607	.	G	A	0/0	0/1	0/1	0/0	PNKP	NON_SYNONYMOUS_CODING	p.Thr92Ile/c.275G>A	dominant	
19	50,789,791	.	G	A	0/0	0/1	0/1	0/0	MYH14	NON_SYNONYMOUS_CODING	p.Arg1490Gln/c.4469G>A	compound recessive and dominant	
19	52,217,060	rs150680444	G	A	0/0	0/1	0/1	0/0	HAS1	NON_SYNONYMOUS_CODING	p.Arg452Trp/c.1354G>A	compound recessive and dominant	
19	58,861,834	.	G	C	0/0	0/1	0/1	0/0	A1BG	NON_SYNONYMOUS_CODING	p.Ser365Cys/c.1094G>C	dominant	YES
19	58,980,635	.	G	A	0/0	0/1	0/1	0/0	ZNF324	NON_SYNONYMOUS_CODING	p.Arg28His/c.83G>A	compound recessive and dominant	
20	30,496,398	rs184016363	G	A	0/0	0/1	0/1	0/0	TTLL9	NON_SYNONYMOUS_CODING	p.Gly21Arg/c.61G>A	dominant	
20	34,785,938	rs144426436	A	G	0/0	0/1	0/1	0/0	EPB41L1	NON_SYNONYMOUS_CODING	p.Lys439Arg/c.1316A>G	dominant	
20	44,516,208	rs143878961	G	T	0/0	0/1	0/1	0/0	SPATA25	NON_SYNONYMOUS_CODING	p.Gln8Lys/c.22G>T	dominant	
20	48,565,831	rs41303809	G	A	0/0	0/1	0/1	0/0	RNF114	NON_SYNONYMOUS_CODING	p.Arg187His/c.560G>A	dominant	
20	62,203,597	rs200635909	T	G	0/0	0/1	0/1	0/0	HELZ2	NON_SYNONYMOUS_CODING	p.Thr48Pro/c.142T>G	dominant	
21	46,354,992	.	C	G	0/0	0/1	0/1	0/0	C21orf67	NON_SYNONYMOUS_CODING	p.Gly117Ala/c.350C>G	dominant	
21	47,532,280	.	G	T	0/0	0/1	0/1	0/0	COL6A2	NON_SYNONYMOUS_CODING	p.Ser168Ile/c.503G>T	dominant	
22	24,717,548	rs56112030	A	T	0/0	0/1	0/1	0/0	SPECC1L	NON_SYNONYMOUS_CODING	p.Leu139Phe/c.417A>T	dominant	
22	25,024,108	rs200266956	G	A	0/0	0/1	0/1	0/0	GGT1	NON_SYNONYMOUS_CODING	p.Arg122Gln/c.365G>A	compound recessive and dominant	
22	29,663,751	rs11539373	G	A	0/0	0/1	0/1	0/0	RHBDD3	START_GAINED		dominant	
