# Supplementary material for: Epistatic interactions between mutations of TACI (TNFRSF13B) and TCF3 result in a severe primary immunodeficiency disorder and systemic lupus erythematosus
Source: Clin Transl Immunology. 2017 Oct 20;6(10):e159–. doi: 10.1038/cti.2017.41 (PMC5671988; doi:10.1038/cti.2017.41)
Supplement: Supplementary Table 3 [file cti201741x4.pdf]

Supplementary Table 3: Genotypes of candidate variants in entire kindred

Gene	Description	Chr	Position	REF	ALT	ExAC ALT allele frequency	Variant classification	I.1	I.2	II.1	II.2	II.3	II.4	III.1	III.2
RGPD3	RANBP2-like and GRIP domain containing 3	2	107,040,309	C	G	8.34E-06	p.Gly1372Arg/missense	C/C	C/C	C/C	C/C	C/C	C/C	C/C	C/C
PLXND1	Plexin D1	3	129,290,644	G	C	3.48E-04	p.Leu1041Val/missense	G/C	G/G	G/G	G/C	G/G	G/C	G/G	G/C
TET2	Tet methylcytosine dioxygenase 2	4	106,111,551	T	C	n/a	-/5-prime-UTR	T/T	T/C	T/T	T/C	T/T	T/C	T/T	T/C
ICE1	Interactor of little elongation complex ELL subunit 1	5	5,463,856	C	T	8.30E-06	p.Ser1470Phe/missense	C/C	T/C	C/C	T/C	T/C	C/C	C/C	T/C
CLLU1	Chronic lymphocytic leukemia up-regulated 1	12	92,818,706	T	C	1.00E-04	p.Tyr84His/missense	T/C	T/T	T/T	T/C	T/C	T/C	T/T	T/C
PLXNC1	Plexin C1	12	94,613,883	A	G	1.01E-03	p.Lys549Arg/missense	A/G	A/A	A/A	A/G	A/G	A/G	A/A	A/G
OASL	2'-5'-oligoadenylate synthetase-like	12	121,458,612	C	T	8.24E-06	p.Glu433Lys/missense	C/T	C/C	C/C	C/T	C/C	C/T	C/C	C/T
TCF3	Transcription factor 3	19	1,623,996	G	GT	n/a	p.Thr168fsX191/frameshift	G/G	G/G	G/G	G/GT	G/G	G/G	G/G	G/GT
VAV1	Vav 1 guanine nucleotide exchange factor	19	58,861,834	G	A	n/a	p.Glu378Lys/missense	G/A	G/G	G/G	G/A	G/G	G/A	G/G	G/A

Chr	Chromosome
Position	Cchromosome position in the GRCh37.p13 reference assembly
REF	Reference sequence allele
ALT	Alternate allele
ExAC ALT AF	Frequency of ALT allele in ExAC (Lek et al. Analysis of protein-coding genetic variation in 60,706 humans. Nature 2016; 536:285), accessed 29 April 2017.
Highlighted	indicating the TCF3 mutation in II.2 and III.1
